# Seasonal phenotype‐specific transcriptional reprogramming during metamorphosis in the European map butterfly *Araschnia levana*

**DOI:** 10.1002/ece3.2120

**Published:** 2016-04-20

**Authors:** Andreas Vilcinskas, Heiko Vogel

**Affiliations:** ^1^ Institute for Insect Biotechnology Justus Liebig University Heinrich Buff Ring 26‐32 35392 Giessen Germany; ^2^ Max‐Planck Institute for Chemical Ecology Hans Knoell Strasse 8 07749 Jena Germany

**Keywords:** *Araschnia levana*, metamorphosis, phenotypic plasticity, polyphenism, transcriptome, wing pattern

## Abstract

The European map butterfly (*Araschnia levana*) is a classic example of seasonal polyphenism because the spring and summer imagoes display two distinct morphological phenotypes. The light regime and temperature during larval and prepupal development determine whether or not the pupae commit to diapause and overwintering and thus whether spring or summer imagoes emerge. We used suppression subtractive hybridization to experimentally screen for genes that are differentially expressed in prepupae committed either to accelerated metamorphosis and egg production or diapause and overwintering. The range and ontology of the differentially expressed genes in prepupae developing from larvae exposed either to long‐day (LD) or short‐day (SD) conditions revealed fundamental differences. The SD prepupae preferentially expressed genes related to cuticle formation and immunity, reflecting the formation of a robust pupal exoskeleton and the upregulation of antimicrobial peptides as preparations for overwintering. One protein preferentially expressed in SD prepupae has a counterpart in *Bombyx mori* that functions as a diapause duration clock. The differentially expressed genes in LD prepupae included several members of the *dusky and osiris* families. We also observed the strong induction of different *yellow*‐like genes under SD and LD conditions which suggest a role in the developmental choice between seasonal phenotypes. Our transcriptomic data will facilitate the more detailed analysis of molecular mechanisms underlying seasonal polyphenism.

## Introduction

Seasonal polyphenism is a phenomenon observed in species that respond to seasonally changing environmental parameters by expressing distinct phenotypes (Shapiro 1976; Simpson et al. 2011). The ability of environmental stimuli to determine which of several phenotypes is expressed by the same genome has led many researchers to investigate the underlying mechanisms (Fric et al. 2004; Suzuki and Nijhout 2008; Daniels et al. 2014).

**Figure 1:**
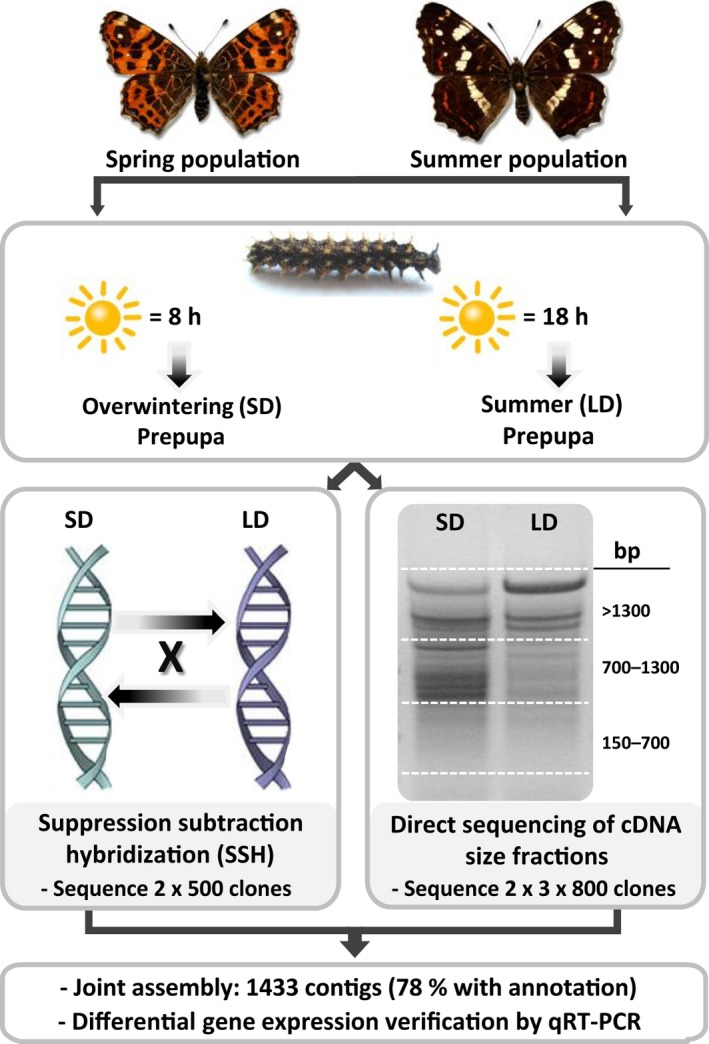
Overview of the experimental design. *Araschnia levana* larvae were exposed to two different light regimes, simulating long‐day (LD, 18‐h photoperiod) or short‐day (SD, 8‐h photoperiod) conditions. Two methods were used to identify differentially expressed genes: suppression subtraction hybridization (SSH, left panel) and direct cloning and sequencing of cDNA size fractions (right panel). The resulting sequences were jointly assembled, annotated, and used for mapping. Primers were designed for selected genes, and the expression patterns were verified by quantitative real‐time RT‐PCR (qRT‐PCR).

The European map butterfly *Araschnia levana* (Linnaeus, 1758) (Lepidoptera: Nymphalidae) is a textbook example of seasonal polyphenism, because it produces imagoes with two strikingly distinct phenotypes (Fig. 1). The spring generation (April to June) is reddish with dorsal black spots (*A. levana levana*), whereas the summer generation (July to August) is dark brown with a white band (*A. levana prorsa*). The summer generation also has a larger body size, wing area, and greater mobility than the spring generation (Morehouse et al. 2013). The caterpillars developing into either the spring or the summer phenotype of the imagoes are exposed to distinct biotic and abiotic environments. Caterpillars encountering long‐day (LD) conditions develop rapidly into adult butterflies of the summer generation (Reinhardt 1984). Their offspring are exposed to short‐day (SD) conditions, and the resulting pupae enter a dormant state known as diapause characterized by enhanced stress tolerance, which is necessary to survive winter temperatures and prolonged exposure to pathogens (MacRae 2010). Both the day length during larval development and the temperature during early pupal development are known to influence the imago phenotype (Reinhardt 1984; Koch 1992; Windig and Lammar 1999). However, little is known about the underlying molecular mechanisms (Fric et al. 2004; Beldade et al. 2011).

We investigated the molecular basis of seasonal polyphenism in *A. levana* by screening for genes that are differentially expressed in prepupae developing from larvae exposed either to LD (18‐h photoperiod) or SD (8‐h photoperiod) conditions. Differentially expressed genes at the onset of metamorphosis were identified by suppression subtractive hybridization (SSH), which has been used successfully for this purpose in several insect species that lack a sequenced genome (Altincicek and Vilcinskas 2007a,b; Vogel et al. 2011; Dobson et al. 2012). Because both the identity of the differentially expressed genes and the direction of differential expression were unknown, we prepared separate cDNA libraries representing prepupae derived from larvae reared under SD or LD conditions and created LD – SD and SD – LD subtracted libraries to identify genes preferentially expressed in the rapidly developing spring phenotype and the overwintering summer phenotype, respectively. We then used a combination of SSH, direct cDNA sequencing, and quantitative real‐time RT‐PCR to identify differentially expressed sequences representing genes with putative diverse roles in the development of *A. levana* seasonal phenotypes.

## Materials and Methods

### Biological specimens

*Araschnia levana* caterpillars were collected in the vicinity of Albach (Germany) either in June (LD) or August (SD). They were fed with stinging nettle cultivars and kept in a climate chamber at 20°C under LD or SD conditions. One day after the onset of pupation, three biological replicates each consisting of five individuals from each group were transferred to vials containing RNAlater (Qiagen, Hilden, Germany) for RNA isolation. The remaining 10 caterpillars under LD conditions developed into the summer generation of butterflies which hatched in August, whereas the remaining 15 caterpillars under SD conditions were allowed to overwinter at 5°C to ensure that the early pupae from both groups were primed appropriately by the light regime to produce distinct phenotypes.

### Suppression subtractive hybridization and sequencing

Total RNA was extracted from whole prepupae developing from caterpillars collected in either June (LD) or August (SD). The RNA samples were extracted using TRI Reagent (Molecular Research Centre, Cincinnati, OH). The integrity of the RNA was verified using an Agilent 2100 Bioanalyzer and a RNA 6000 Nano Kit (Agilent Technologies, Palo Alto, CA). The quantity of RNA was determined using a Nanodrop ND‐1000 UV/Vis spectrophotometer (Thermo Fisher Scientific, Waltham, MA). Poly(A)+ mRNA was isolated using the MN‐NucleoTrap mRNA kit according to the manufacturer's instructions (Macherey & Nagel, Düren, Germany). SSH was carried out on prepupal mRNA from the LD and SD samples using the SMART PCR cDNA synthesis kit (Clontech, Mountain View, CA) and the PCR‐Select cDNA subtraction kit (Clontech) as previously described (Altincicek and Vilcinskas 2009; Vogel et al. 2011). The RNA was pooled from all 15 prepupae in each treatment group for analysis. SSH was carried out in both directions, using either LD or SD mRNA as the driver. The subtraction efficiency of the cDNA library was confirmed using 1 ng of nonsubtracted and subtracted cDNA to amplify the housekeeping gene *α‐tubulin*. Fractions of the resulting SD – LD and LD – SD subtracted cDNA pools were cloned in the pCRII‐TOPO vector and introduced into *Escherichia coli* ELECTROMAX DH5α‐E electrocompetent cells (Invitrogen, Carlsbad, CA) to create a pair of bidirectional subtracted libraries. We then transferred 500 bacterial colonies from each library into 96‐deep‐well plates and isolated plasmid DNA using the 96‐well robot plasmid isolation kit (NextTec Biotechnologie GmbH, Hilgertshausen, Germany) on a Tecan Evo Freedom 150 robotic platform (Tecan, Männedorf, Switzerland). Nonsubtracted cDNA produced from the LD and SD mRNA pools was resolved by agarose gel electrophoresis and extracted from the gel as three different size fractions (150–700, 700–1300, and >1300 bp) in order to improve cloning efficiency for larger cDNAs. Each size fraction of the LD and SD pools was purified, cloned in the pCRII‐TOPO vector, and introduced into *E. coli* cells as above to create six size‐fractionated nonsubtracted libraries. We then transferred 800 colonies from each library into microtiter plates for plasmid preparation as described above. The 5′ and 3′ termini of the subtracted and nonsubtracted cDNA library clones were sequenced on an ABI 3730 xl automatic DNA sequencer (PE Applied Biosystems, Foster City, CA). Vector clipping, quality trimming, and sequence assembly under stringent conditions (e.g., high‐quality sequence trimming parameters, 95% sequence identity cutoff, 25‐bp overlap) with the individual sequence trace files were carried out using the Lasergene software package (DNAStar Inc., Madison, WI).

The resulting sequences were used to search the National Center for Biotechnology Information (NCBI) database with the blastall program. Homology searches (BLASTx and BLASTn), and functional annotation according to gene ontology (GO) terms (http://www.geneontology.org), InterPro terms (InterProScan, EBI), enzyme classification (EC) codes, and metabolic pathways (Kyoto Encyclopedia of Genes and Genomes, KEGG) were carried out using BLAST2GO v2.3.1 (http://www.blast2go.de) (Conesa & Götz 2008). Homology searches were conducted remotely on the NCBI server with QBLAST using a sequential strategy. First, sequences were searched against the NCBI nonredundant (nr) protein database using an E‐value cutoff of 10^−3^, with predicted polypeptides of a minimum length of 15 amino acids. Second, sequences that did not retrieve BLASTx hits were searched again using BLASTn, against the NCBI nr nucleotide database, using an E‐value cutoff of 10^−10^. The GO data represent the level 3 analysis, illustrating general functional categories. Enzyme classification codes and KEGG metabolic pathway annotations were generated from the direct mapping of GO terms to their enzyme code equivalents. Finally, InterPro searches were carried out remotely using BLAST2GO via the InterProEBI web server. An assembly of the complete *A. levana* dataset with contig consensus sequences, BLAST2GO hits against the NCBI nr nucleotide database, hit accessions, annotations including InterPro scans and maps of the individual sequences from all four sample groups (LD – SD SSH, SD – LD SSH, and LD and SD direct sequencing) to the combined and assembled contig sequences can be found in Supporting Information File 1. Individual reads were mapped to contigs using CLC Genomics Workbench v7.1 (http://www.clcbio.com). Fisher's exact test was used as part of BLAST2GO to look for the overrepresentation of GO terms among lists of identified genes in the LD – SD and SD – LD subtracted datasets relative to the complete reference dataset, which contained the assembly of all sequences obtained by SSH and direct sequencing, whereas the test sets comprised either the LD – SD SSH dataset (containing genes more abundant in SD prepupae) or the SD – LD SSH dataset (containing genes more abundant in LD prepupae). The GO‐enriched bar charts were simplified to display only the most specific GO terms by removing parent terms representing existing child terms using the function “Reduce to most specific terms” implemented in BLAST2GO. A GO term was considered significantly enriched if the *P*‐value corrected by false discovery rate control (FDR) was <0.05.

### Quantitative real‐time RT‐PCR analysis

The differential expression of a selected set of expressed sequence tags (ESTs) identified by SSH and the direct sequencing approach was confirmed by quantitative real‐time RT‐PCR using primers based on the EST data (Table S1) and designed using Primer3Plus (http://primer3plus.com/cgi-bin/dev/primer3plus.cgi). The ESTs selected for primer design and subsequent expression verification by qPCR were chosen based on their putative functions which could relate to the observed differences between LD and SD prepupae, such as cuticle formation, innate immunity, hormonal signaling, molting, or the diapause. Primers were tested by generating individual melting curves and by preparing dilution series for primer efficiency calculations. Primers with a single peak in the melting curve and an efficiency value between 0.9 and 1.1 were used in subsequent qPCR experiments. Each sample (biological replicate) was measured in duplicate (technical replicates), and mean values of these duplicates (in case the differences in Ct values did not exceed 0.2) were used for relative quantification using the comparative Ct method. Reverse transcription and real‐time PCR were carried out on three biological replicates of the LD and SD samples using the FullVelocity SYBR^®^ Green QRT‐PCR kit (Stratagene, La Jolla, CA) on the Mx3000P system (Stratagene) according to the manufacturer's recommendations. We considered four different reference genes at the beginning of the qPCR experiments, namely elongation factor 1‐alpha (*EF1α*), ribosomal protein L10 (*RPL10*), ribosomal protein S8 (*RPS8*), and eukaryotic initiation factor 4‐alpha (*EIF4α*). These genes were selected based on their status as stable reference genes in other insects as well as our own experience with these genes as suitable reference genes in a larger number of nonmodel insect species. We used the program NormFinder (www.mdl.dk) to identify the most stable reference genes among the four genes we tested. Normfinder ranks the set of candidate normalization genes according to their expression stability in a given sample set and given experimental design. The gene with the most stable expression (defined as the candidate with the smallest intergroup variation and errors bars) was *EF1α*. Our data were thus normalized against *EF1α*, an internal control which has recently been shown to allow accurate normalization in different insect tissues (Baumann et al. 2015). The ΔCT values for the SD samples were calibrated against the LD samples and vice versa. The ΔΔCT value was then used to calculate the fold difference in expression between the SD and LD pupae. Statistical analysis of fold‐change differences between LD and SD prepupal samples was performed using a *t*‐test with all individual log (base2)‐transformed delta Cq values. *P*‐values <0.01 were considered to be significant.

## Results

We investigated differences in gene expression between *A. levana* prepupae derived from larvae exposed to LD or SD conditions to identify candidate genes involved in seasonal polyphenism. We created mRNA pools from pupae in the LD and SD experimental groups and carried out bidirectional SSH to create LD – SD and SD – LD subtracted cDNA pools enriched for differentially expressed sequences. We also used the original LD and SD mRNA pools to produce nonsubtracted cDNA. Side‐by‐side comparison of the nonsubtracted cDNA pools by agarose gel electrophoresis revealed striking differences in the LD and SD banding patterns, suggesting differences in the expression of a large number of genes (Fig. 1). The control caterpillars reared under LD or SD conditions developed into the corresponding distinct phenotypes (the summer morph hatched in August whereas the spring morph hatched after overwintering).

We divided the gel into three horizontal layers representing different cDNA size fractions (150–700, 700–1300, and >1300 bp) and extracted the cDNA separately from each fraction to yield six subpools which were used to create cDNA libraries. We picked 800 random clones from each of the six libraries for sequencing and analysis. Similarly, the LD – SD and SD – LD subtraction pools were also used to prepare cDNA libraries and we picked 500 random clones from each library for sequencing and analysis. The sequences of both the direct sequencing and the SSH approach were jointly assembled, and individual samples were remapped to the resulting partial transcriptome (Table S1). When we compared the mapping results of the direct sequencing and the SSH data, 16 of the SSH‐derived ESTs induced in SD prepupae showed a similar profile in the sequencing dataset, that is, they were only present in the SD prepupae sample used for direct sequencing. Similarly, 22 ESTs derived from the reverse SSH experiment, enriching for genes induced in the LD prepupae, were only present in the LD prepupae sample used for direct sequencing. The ESTs for which differential expression between SD and LD prepupae could be confirmed by both SSH and direct sequencing, included overexpressed sequences encoding cuticle proteins and methionine‐rich storage proteins. Thus, although we confirmed a proportion of the more strongly expressed SSH‐derived ESTs by direct sequencing, the majority of the SSH‐derived ESTs were not matched to reads from the direct sequencing approach.

**Figure 2:**
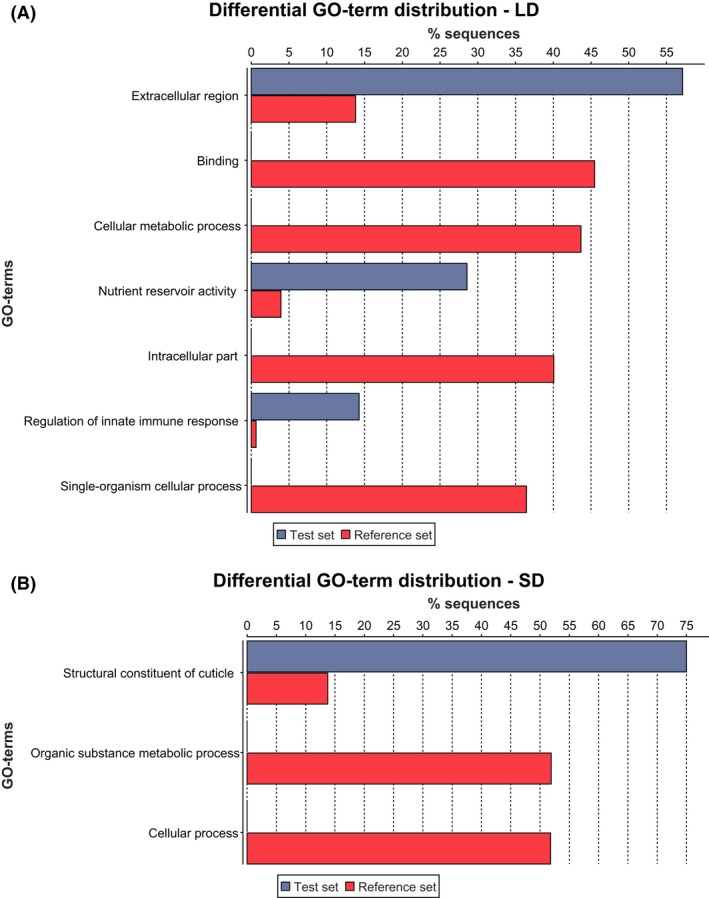
Differential distribution of Gene Ontology (GO) terms. Bar charts show the GO terms that were significantly (false discovery rate (FDR) <0.05) enriched in the long‐day (LD) and short‐day (SD) libraries, respectively. The GO terms are sorted in an ascending order according to their FDR value, starting with the most significantly enriched. Only the most specific GO terms are displayed. Differences are shown as the percentage of sequences associated with a specific GO category in the test set (total number of differentially expressed contigs between LD prepupae (A) and SD prepupae (B) versus the reference set (transcriptome backbone assembly) using Fisher's exact test in BLAST2GO‐PRO.

**Figure 3:**
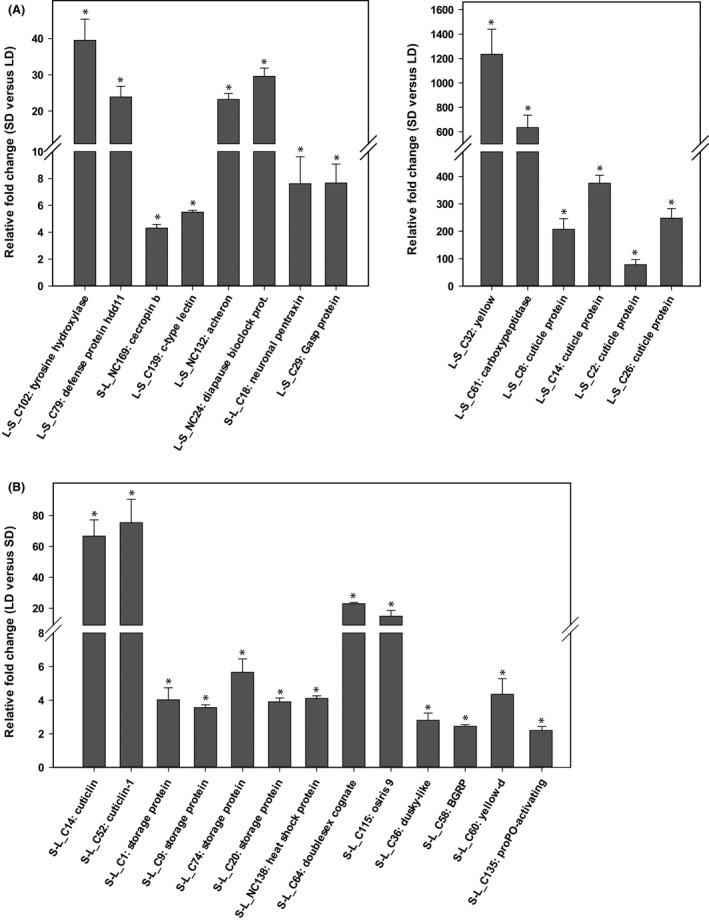
Quantitative real‐time RT‐PCR results for candidate genes. (A) Relative expression levels of selected genes preferentially expressed in short‐day (SD) prepupae, determined by quantitative real‐time RT‐PCR. (B) Relative expression levels of selected genes preferentially expressed in long‐day (LD) prepupae, determined by quantitative real‐time RT‐PCR. Expression levels were normalized to *EF1α* and presented as relative fold changes (mean ± SD, *n* = 3). Statistically significant differences (*P* < 0.01) are marked with an asterisk.

The ESTs in each subtraction library were analyzed in more detail by identifying enriched GO terms using Fisher's exact test. This revealed substantial differences in the representation of genes in the LD and SD experimental groups (Fig. 2). Prepupae derived from the SD larvae (committed to diapause and overwintering) preferentially expressed genes encoding cuticle‐related proteins whereas prepupae derived from the LD larvae (committed to accelerated metamorphosis) preferentially expressed genes related to nutrient reservoir activity and the regulation of innate immunity (Fig. 2). The differential expression of 72 ESTs representing cuticle formation, innate immunity, hormonal signaling, molting, or the diapause was confirmed by quantitative real‐time RT‐PCR (Fig. 3) using the primers listed in Table S2. Among a total of 72 genes tested by quantitative real‐time RT‐PCR, four primer combinations did not meet our quality criteria (see methods for details) and another four resulted in very high Ct values and were not taken into account for fold‐change calculations. Among the remaining 64 genes, 50 displayed statistically significant differences in expression (*P* < 0.01) between the SD and LD samples according to the SSH data. Quantitative real‐time RT‐PCR results for another 14 genes were either nonsignificant or did not show differential expression between the SD and LD prepupae.

Among the transcripts upregulated under SD conditions, those related to cuticle formation were the most abundant (Fig. 2). This was confirmed by quantitative real‐time RT‐PCR for four genes encoding cuticle proteins as well as several others related to cuticle development and defense functions (Fig. 3A). We also identified *A. levana* orthologs of the developmental regulator Acheron and the *Bombyx mori* diapause bioclock protein (time interval measuring enzyme esterase A4), both of which were strongly induced under SD conditions (Fig. 3A).

In contrast, LD conditions induced several genes encoding methionine‐rich storage proteins, as well as heat‐shock protein 70, a chitin deacetylase, at least one member of the lipocalin family of transporters, a prophenoloxidase‐activating factor, and a methyltransferase (Fig. 3B). Several members of the *dusky, osiris,* and *yellow* gene families were also identified among the genes induced under LD conditions, each of which has been implicated in the control of developmental processes including pigmentation (Fig. 3B). Another member of the *yellow* family was strongly induced under SD conditions (Fig. 3A) suggesting yellow proteins may play a role in the developmental choice between the alternative seasonal phenotypes.

## Discussion

Environmental stimuli can influence the choice between alternative phenotypes in many insect species, but the underlying molecular mechanisms remain largely uncharacterized (Fric et al. 2004; Suzuki and Nijhout 2008; Beldade et al. 2011). We used SSH and cDNA sequencing to study differences in gene expression between *A. levana* prepupae derived from larvae collected and reared under LD or SD conditions, aiming to identify candidate genes involved in seasonal polyphenism. SSH is a useful method for the identification of differentially expressed genes in the absence of a complete genome sequence because it allows the isolation of both upregulated and downregulated genes even when the number and nature of the genes and their direction of change are unknown (Altincicek and Vilcinskas 2007a,b; Vogel et al. 2011; Dobson et al. 2012). It combines normalization and suppression PCR steps in a single cycle and can achieve a high level of enrichment for rare, differentially expressed transcripts (up to 5000‐fold). However, depending on the experimental procedure, SSH cannot exclude all commonly expressed genes, resulting in the isolation of false positive clones. We validated the positive clones identified by either direct sequencing or the SSH approach by confirming the differential expression of the candidate genes by quantitative real‐time RT‐PCR. This approach confirmed 80% of the differences in expression between the SD and LD prepupae.

Furthermore, the SSH approach only allows the direct comparison of two samples (Diatchenko et al. 1996; Huang et al. 2007). RNA‐Seq does not have this limitation, but like the SSH approach, it can combine de novo transcriptome generation and differential expression analysis for nonmodel organisms. However, the sensitivity and accuracy of RNA‐Seq largely depends on the millions of reads sequenced per sample, the number of replicates used and the filtering and mapping procedures for data processing. For example, in a study comparing RNA‐Seq sequencing depth and the identification of differentially expressed genes, 10 million mapped fragments were sufficient to confirm the differential expression of the most strongly expressed genes but genes with lower expression levels suffered a high FDR (SEQC/MAQC‐III Consortium 2014). Therefore, although the SSH method can yield false positives, the identification of differentially expressed genes with low expression levels by RNA‐Seq requires a greater sequencing depth, and this is more expensive.

By comparing nonsubtracted cDNA pools in three size fractions, we observed striking differences in the pattern of bands displayed on agarose gels, suggesting that *A. levana* seasonal polyphenism involves profound differences in the expression of a large number of genes (Fig. 1). In more general terms, when we compared the direct sequencing and SSH data, a number of ESTs that are differentially expressed in SD and LD prepupae could be confirmed by both methods, including methionine‐rich storage proteins and cuticle proteins.

The ESTs in each subtraction library were screened for enriched GO terms revealing striking differences in the representation of genes in the LD and SD groups. Prepupae derived from the SD larvae (committed to diapause and overwintering) preferentially expressed genes encoding cuticle‐related proteins, whereas prepupae derived from the LD larvae (committed to accelerated metamorphosis and egg production) preferentially expressed genes related to nutrient reservoir activity and the regulation of innate immunity (Fig. 2). The transcripts upregulated under SD conditions included many involved in cuticle formation, presumably because SD conditions promote the development of a thicker and more robust exoskeleton for pupae committed to diapause and overwintering (Stuckas et al. 2014). The greater abundance of cuticle proteins allows the development of a stiffer and less permeable cuticle to prevent water loss and promote survival (Baker and Russell 2009; Li and Denlinger 2009). SD conditions also induced at least one member of the Gasp/Obstructor family of chitin‐binding proteins, which are localized mainly in cuticle‐forming tissues where they control epithelial extracellular matrix dynamics, cuticle integrity, and exocuticle formation (Behr and Hoch 2005; Nisole et al. 2010).

The hardening of the cuticle involves the cross‐linking of cuticle proteins (sclerotization), and this requires dopamine derivatives produced by the enzymes tyrosine hydroxylase and DOPA decarboxylase (Hiruma and Riddiford 2009). Accordingly, genes encoding both enzymes were preferentially expressed by the SD prepupae, and the differential expression of tyrosine hydroxylase mRNA was confirmed by quantitative real‐time RT‐PCR (Fig. 3A). The SD prepupae also expressed high levels of a transcript encoding an Acheron‐like protein (Fig. 3A). The *Manduca sexta* ortholog is an RNA‐binding protein that regulates apoptosis during the development of skeletal muscles (Valavanis et al. 2007). The SD prepupae also preferentially expressed genes encoding immunity‐related factors such as a c‐type lectin acting as a pattern recognition receptor and the antimicrobial peptide cecropin B (Fig. 3A). This induction of immunity‐related genes reflects the preparation for diapause and overwintering, during which the pupae are more vulnerable to microbial infection (Nakamura et al. 2011).

LD conditions induced a number of genes encoding methionine‐rich storage proteins, which function as precursor reservoirs for reproduction and metamorphosis in other lepidopteran insects (Pan and Telfer 1996). The proteins accumulate in last‐instar larvae and promote egg development, as expected for a seasonal phenotype prepared for imminent reproduction (Ashfaq et al. 2007; Damara and Dutta‐Gupta 2010; Sonoda et al. 2006). The induction of several genes encoding storage proteins was confirmed by quantitative real‐time RT‐PCR (Fig. 3B). Heat‐shock protein 70 was also induced under LD conditions, consistent with its role before, during, and after diapause (Bahar et al. 2007).

The most striking discovery among our panel of differentially expressed genes was an *A. levana* ortholog of the *B. mori* diapause bioclock protein (time interval measuring enzyme esterase A4), an ATPase with a copper–zinc superoxide dismutase domain (Isobe et al. 2006). The identification of a differentially expressed ATPase, which is known to be responsible for measuring the duration of diapause in *B. mori* eggs, provides a plausible mechanism to explain how the enhanced expression of the *A. levana* homolog under SD conditions (Fig. 3A) may contribute to the induction of diapause in the resulting pupae, which produce the spring morph following prolonged pupal development. The lower expression of this ATPase in *A. levana* under LD conditions (Fig. 3A) may reflect the fact that these prepupae are not committed to diapause and therefore do not require a molecular clock that measures its duration.

LD conditions also induced the expression of chitin deacetylase, which was previously identified as one of three proteins present specifically in the non‐diapausing pupae of *Helicoverpa armigera* (Chen et al. 2010). The LD prepupae also preferentially expressed at least one member of the lipocalin family of transporters, which carry small hydrophobic molecules such as steroids and lipids, and may play a role in insect coloration, immunity, olfaction and pheromone transport (Flower 1996). We also found that LD prepupae expressed higher levels of prophenoloxidase‐activating factor, which presumably activates the prophenoloxidase required for melanin formation in the darker summer phenotype. The upregulation of a methyltransferase under LD conditions suggests that the transcriptional reprogramming that accompanies seasonal polyphenism may be regulated by epigenetic mechanisms, as we recently confirmed in another lepidopteran species (Mukherjee et al. 2012; Mukherjee and Vilcinskas 2014).

Several members of the *dusky, osiris,* and *yellow* gene families were also identified among the genes induced under LD conditions. Dusky is a family of zinc finger proteins which control cytoskeletal reorganization during wing morphogenesis and cuticle formation in bristles of the fruit fly *Drosophila melanogaster* (Roch et al. 2003; Nagaraj and Adler 2012). The *osiris* gene family arose by duplication and neo‐functionalization soon after the divergence of insects from other arthropods (Shah et al. 2012). Although the function of Osiris proteins is unclear, they contain signal peptides and transmembrane domains suggesting they project into the extracellular environment, and their dynamic expression profiles in *D. melanogaster* larvae suggest a role in development (Chintapalli et al. 2007). Yellow‐like proteins are found in bacteria and insects, and in the latter, they play a role in pigmentation and wing patterning, but their molecular role is largely unknown (Ferguson et al. 2011). The strong induction of different *A. levana yellow*‐like genes under SD and LD conditions suggests a role in the developmental choice between the seasonal phenotypes, which is supported by recent experiments showing seasonal‐specific differential expression of *yellow*‐like genes in the fifth‐instar larvae of the buckeye butterfly *Junonia coenia*, which also occurs in distinct seasonal phenotypes (Daniels et al. 2014). The gender‐specific expression of a *yellow* gene in an ant regulates sex‐specific melanin synthesis resulting in a dimorphic body color (Miyazaki et al. 2014). Therefore, we postulate that differentially expressed *yellow* genes in *A. levana* may also influence the distinct coloration of the seasonally occurring adult morphs. The *yellow‐e* gene has recently been shown to protect *Tribolium castaneum* against dehydration (My et al. 2015). The upregulation of corresponding genes in *A. levana* prepupae committed to diapause may also enhance waterproofing during the winter.

In conclusion, our experimental screen for differentially expressed genes in *A. levana* prepupae derived from caterpillars exposed to either LD or SD conditions revealed profound differences at the level of the transcriptome. The number of differentially expressed genes and the complexity of the pathways and gene ontologies suggest that seasonal polyphenism in *A. levana* involves a broad reprogramming of the transcriptome in response to day length. LD conditions induce genes encoding storage proteins, which are required for egg production and rapid development into adult butterflies, whereas SD conditions induce genes representing cuticle development, innate immunity, and a putative diapause bioclock protein. Members of the *dusky*,* osiris,* and *yellow* gene families are also differentially regulated, representing potential mediators of epigenetic regulation and the control of pigmentation. Our data provide a basis to explore in more detail the molecular mechanisms of seasonal polyphenism in lepidopteran insects. For example, the differentially expressed genes will be silenced by RNA interference to investigate their role in the determination of seasonal phenotypes.

## Data Accessibility

An assembly of the complete *A. levana* dataset with contig consensus sequences, BLAST2GO hits against the NCBI nr database, hit accessions, and annotations including InterPro scans can be found in Supporting Information File 1.

## Conflict of Interest

None declared.

## Supporting information


**Table S1.** Sequences, annotation and mapping data for *Araschnia levana* contigs derived from SSH and direct sequencing.

**Table S2.** Primer sequences and qRT‐PCR expression data for Araschnia levana contigs derived from SSH and direct sequencing.
